# Real-Time Exposure to Intersectional Minority Stressors and Alcohol Use: Protocol for an Ecological Momentary Assessment Study With Latinx and Non-Latinx Sexual Minority Youth

**DOI:** 10.2196/87201

**Published:** 2026-01-30

**Authors:** Robert Rosales, Suzanne M Colby, Kristina M Jackson, Christina S Lee, Jacob John van den Berg, Madelyn Clancy, Ethan H Mereish, Robert Miranda Jr

**Affiliations:** 1 Center for Alcohol and Addiction Studies; Center for Health Promotion and Health Equity Department of Behavioral and Social Sciences Brown University Providence, RI United States; 2 Center for Alcohol and Addiction Studies Department of Behavioral and Social Sciences; Department of Psychiatry and Human Behavior Brown University Providence, RI United States; 3 Rutgers Robert Wood Johnson Medical School Rutgers Addiction Research Center, Department of Psychiatry Rutgers, The State University of New Jersey Piscataway, NJ United States; 4 School of Social Work Boston University Boston, MA United States; 5 Department of Psychology and Education College of Arts and Sciences Norwich University Northfield, VT United States; 6 School of Public Health Center for Alcohol and Addiction Studies Brown University Providence, RI United States; 7 Department of Psychology University of Maryland, College Park College Park, MD United States

**Keywords:** Latinx, sexual minority youth, alcohol, minority stress, intersectionality, ecological momentary assessment

## Abstract

**Background:**

Sexual minority youth (SMY) are significantly more likely to use alcohol compared with their heterosexual peers. Recent national data also suggest a turning point in alcohol use disparities: Latinx youth now report higher alcohol use than non-Latinx youth. Despite this, little is known about the social context and reasons why Latinx SMY may engage in alcohol use.

**Objective:**

This manuscript describes the protocol for a study designed to assess real-time exposure to minority stressors and protective factors, and their relationship to alcohol use among Latinx and non-Latinx White SMY.

**Methods:**

The project is being conducted in 3 phases with a combined sample of approximately 140 participants. Phase 1 (completed) involved cognitive interviews with 23 SMY participants, which refined and adapted survey measures to ensure cultural and developmental appropriateness for the next study phases. Phase 2 was a pilot ecological momentary assessment (EMA) survey with 20 participants to evaluate feasibility, acceptability, and compliance (completed). Phase 3 will recruit approximately 100 SMY aged 15-19 years, with equal representation of Latinx and non-Latinx White SMY. Participants will complete a baseline survey and repeated EMA surveys to capture daily experiences of stressors, protective factors, and alcohol use.

**Results:**

Results for the study sample, recruitment (between April 2022 and November 2023), and challenges confronted are presented for Phase 1. Findings showed that the study sample included 23 Latinx and non-Latinx SMY, split almost in half by ethnicity. Participants were mostly female and affluent. Recruitment efforts showed that certain flyers and locations (eg, Facebook/Instagram) performed better at recruiting this sample. We present issues faced with screening out ineligible participants and bots, recruiting participants assigned male at birth, recruiting 15- to 17-year-old participants, and building overall trust with this population. Results from the rest of the data in this study will be analyzed and disseminated through peer-reviewed scientific journals.

**Conclusions:**

This study will provide novel data on the real-time contexts of alcohol use among SMY with particular attention to Latinx youth, an understudied and marginalized population. By identifying stress and protective mechanisms linked to alcohol use, findings can inform tailored prevention and intervention strategies. Furthermore<strong>,</strong> the protocol offers a replicable framework for future EMA research on intersectionality, minority stress, and alcohol use among diverse SMY populations.

**International Registered Report Identifier (IRRID):**

DERR1-10.2196/87201

## Introduction

### Overview

Underage alcohol use is a significant public health concern in the United States, with particularly heightened risks among sexual minority youth (SMY). Data from the 2021 Youth Risk Behavior Surveillance Survey, a nationally representative survey of middle and high school youth, show that nearly half of adolescents have consumed alcohol in their lifetime, and approximately 30% drank in the past 30 days [1]. SMY are at higher risk of alcohol use and related problems compared with heterosexual peers [2-4]. SMY also tend to initiate drinking at earlier ages, and more frequently progress to alcohol use disorders [5]. These disparities may be partly attributable to the stress associated with heterosexism and other forms of discrimination [6].

Data from the Monitoring the Future study suggest that Latinx youth may now be more likely than non-Latinx White youth to consume alcohol and engage in high-risk drinking behaviors [7,8]. Longitudinal national data further suggest that discrimination may contribute to this increase in alcohol use among Latinx youth [9]. Despite these concerning trends, a recent review emphasized substantial gaps in the literature, noting that little is currently known about the risk and protective factors predicting substance use among Latinx SMY [10].

Minority stress theory (MST) posits that sexual minority individuals may use alcohol as a means of coping with negative affect arising from experiences of oppression-based stress [11-16]. MST further emphasizes the importance of social context, noting that the setting in which minority stress occurs—such as at school or at home—can be a critical predictor of substance use [17]. However, evidence on alcohol, tobacco, and other drugs (ATOD) use among Latinx SMY, compared with White SMY, remains mixed. Some studies report higher rates of ATOD use among Latinx SMY [18,19], whereas others indicate lower rates relative to White SMY [20]. To explain these inconsistencies, MST offers 2 competing hypotheses. The resilience hypothesis posits that exposure to racism may foster resilience to heterosexism, potentially buffering Latinx SMY from substance use risk compared with White SMY [6]. Support for this hypothesis comes from research on Latinx youth more broadly, which highlights unique sociocultural resilience factors. For example, ethnic identity may serve as a protective resource, mitigating the additive stress associated with discrimination based on cultural values and language [21]. Aligned with Intersectional Theory, the double jeopardy hypothesis suggests that Latinx SMY may face an elevated risk of ATOD misuse due to the compounded stress of occupying both sexual and ethno-racial minority statuses [6]. However, research suggests that the effects of minority stress on ATOD use among this population can also be explained by momentary changes in affect.

### Affect and Alcohol Use

Affect refers to subjective emotional states, typically categorized as positive or negative [22,23]. Although findings are mixed, prior research has demonstrated that negative affect is correlated with greater substance use, whereas positive affect tends to be associated with lower levels of use [24]. One proposed mechanism is that individuals may use substances to reduce or regulate negative emotions [25]. Negative emotional experiences have been identified as risk factors for substance use among minority populations, including sexual minority and Latinx youth [4,26]. Such negative emotions are often linked to minority-related stressors, including the challenges of coming out and experiences of discrimination [4,27]. Importantly, recent ecological momentary assessment (EMA) research has shown that negative affect can mediate the relationship between minority stress and substance use (specifically tobacco use) among SMY [28]. However, considerably less is known about the role of ethnicity in these processes, including whether there are sociocultural protective factors that may buffer the impact of minority stress on ATOD use.

### Study Objective

This paper outlines the protocol for a study designed to examine the relationships among intersectional minority stress, protective factors, affect (both positive and negative), and alcohol use in the natural environment between Latinx and non-Latinx White SMY (ages 15-19 years). Although White SMY experience similar experiences with minority stress for their sexual orientation, Latinx SMY may face additional stress because of having to deal with cultural differences, language discrimination, and xenophobia in the United States. High school students aged 15 to 19 years are at a transition age where they are developing their identity and forming an independent relationship with their parents. This independence also puts them at greater risk of being influenced by their peers, who may discriminate against them, before they have developed all their coping mechanisms [29].

The overarching goal is to address gaps in the literature on alcohol use risk within this population and to identify protective mechanisms that may be leveraged in intervention [10]. This study protocol consists of three phases: (1) a qualitative phase to explore minority stress and alcohol use and to refine/adapt measures for the EMA phase, (2) a pilot study with follow-up interviews to assess the feasibility and acceptability of the survey, and (3) a 21-day EMA protocol. Participants in both the pilot and full EMA phases (2 and 3) also complete an extensive baseline survey to assess control and moderating variables.

EMA is a data collection approach that captures experiences as they occur, ideally reducing recall bias and enhancing ecological validity [30]. In this study, EMA is used to address the overarching research aim of examining the additional stress burden experienced by Latinx SMY due to daily experiences of minority stress, when compared with White SMY. EMA methods make it possible to assess attitudes and behaviors related to ATOD use in real-time, including momentary affect, alcohol use episodes, and contextual risk and protective factors as youth encounter minority stress throughout the day [31,32]. The repeated measures design of EMA allows us to evaluate whether minority stress and affect are contemporaneously associated with alcohol use (eg, within the same day or week). In addition, the contextual risk and protective factors at the individual, family, and environmental levels are assessed in real time. Participants report when, where, and with whom minority stress occurred, along with their perceptions of whether these experiences are related to alcohol use.

This protocol paper presents (1) the study methods for all 3 phases, including design, procedures, compensation, measures, and analytic approach; (2) sample description and lessons learned from the first phase of the study related to recruitment strategies; and (3) the strategies used to address challenges during implementation of Phase 1. The purpose of providing this information is to offer a roadmap that may help future researchers navigate the complexities of conducting research with this population more efficiently and with fewer obstacles.

## Methods

### Design

The study protocol was organized into 3 phases. The targeted combined sample of 140 Latinx and non-Latinx White SMY ensures equal representation of Latinx and non-Latinx White participants in each phase. The target sample of 140 participants is not powered to find between-person differences in alcohol use. Thus, this study will provide the feasibility and acceptability of this protocol for future fully powered EMA studies on this topic. It will also serve as preliminary findings showing potential signals for between-person and within-person differences in alcohol use. Across all phases, participants are recruited according to the same study inclusion and exclusion criteria. Eligibility criteria are (1) self-identify as a sexual minority with one or more sexual orientation dimensions (same-sex behavior, attraction, or identity) [33]; (2) identify as either Hispanic/Latinx (any race) or non-Hispanic/Latinx White; (3) between the ages of 15 and 19 years; (4) report at least one minority stressor (ethnic/racial or sexual, such as victimization or discrimination) within the past 30 days; (5) report alcohol use within the past 30 days; (6) speak English or Spanish; (7) currently reside in the United States; and (8) have access to a phone or other device capable of completing daily surveys and interviews.

### Ethical Considerations

All study procedures were reviewed and approved by the Brown University Institutional Review Board (IRB; protocol numbers 556 (Phase 1), 383 (Phases 2 and 3).

Across all study phases, informed consent (or assent for minors) was obtained either by having participants read the consent form themselves or by having a research team member read it aloud. The consent process was designed to ensure comprehension and voluntariness. The purpose and procedures of the study were presented in clear, accessible language, available in both English and Spanish. Potential participants were given adequate time to consider their decision, had the opportunity to ask questions, and were informed that participation was voluntary. Because all participants were required to have functional proficiency in English or Spanish, translation services were not necessary. The principal investigator and most research team members were fluent in both languages and able to conduct the process bilingually. All participants were provided with contact information for study staff and encouraged to reach out with any questions. It was emphasized that all survey information would remain confidential.

Prior research has documented the potential for psychological or physical harm to youth who disclose their sexual orientation to parents [34,35]. In addition, studies indicate that many SMY decline to participate in research requiring parental consent, thereby increasing self-selection bias [36-38]. Such bias threatens the validity of findings and may limit the applicability of recommendations for this population. The Institute of Medicine has similarly noted that “certain studies that are important to adolescent health and well-being will not be feasible without such a waiver [of parental consent]” [39]. Given these considerations—and the minimal risks associated with participation—the IRB determined that parental consent was not appropriate for this study. The waiver of parental consent was granted in accordance with Brown University’s IRB requirements, which stipulate that (1) the research involves no more than minimal risk to participants, (2) the waiver does not adversely affect participants’ rights or welfare, and (3) the research could not practicably be conducted without the waiver. The IRB approved a waiver of documentation of consent, allowing participants aged 18 years and older to provide verbal consent and participants aged 15-17 years to provide verbal assent without parental permission.

Since this is not a clinical trial, the IRB also approved that we do not access EMA data in real time. The data are only reviewed at the end of the study or when all data are collected. Given that the focus is on underage alcohol use in a marginalized population, we also provide participants with a behavioral health resource document that provides them with contacts for national and local behavioral health organizations that can be contacted through phone call or text during an emergency. All data was deidentified before the data analytic process.

### Recruitment

The study used the same recruitment strategies across all phases (target sample size=140). Recruitment efforts were multi-pronged. First, printed materials (eg, posters and brochures) were distributed, with organizational approval, to community-based settings nationwide (eg, Los Angeles, California; Boston, Massachusetts; Providence, Rhode Island; and Phoenix, Arizona), including high schools, colleges and universities, health centers, libraries, Latinx-serving organizations, and organizations serving SMY youth. Second, study staff continued outreach at community events such as Pride celebrations and Latinx cultural gatherings in the northeastern US, as well as in high-traffic areas (eg, bus stops, convenience stores, markets, and laundromats). Third, targeted digital recruitment was implemented through targeted ads (by age and location) on social media platforms (eg, Facebook and Instagram) to enhance reach to SMY populations throughout all study phases. All recruitment materials were available in both Spanish and English.

All recruitment flyers contained standardized information. Each flyer included (1) a call to study action (eg, “Help your community!”), (2) eligibility criteria regarding age (eg, “Are you a teenager 15-19 years old?”), (3) a general study overview (eg, “We invite you to join a voluntary research study on stress and health”), (4) a description of study procedures (eg, “The study is fully remote and will involve two interviews and completion of phone-based surveys over three weeks”), (5) information about compensation (eg, “You can receive up to $200 for participating”), (6) instructions for next steps (eg, “To check eligibility, scan the QR code or complete a brief survey”), and (7) study team identifiers (eg, Brown University logo, study phone number, email address, website, and IRB protocol number).

**Figure 1:**
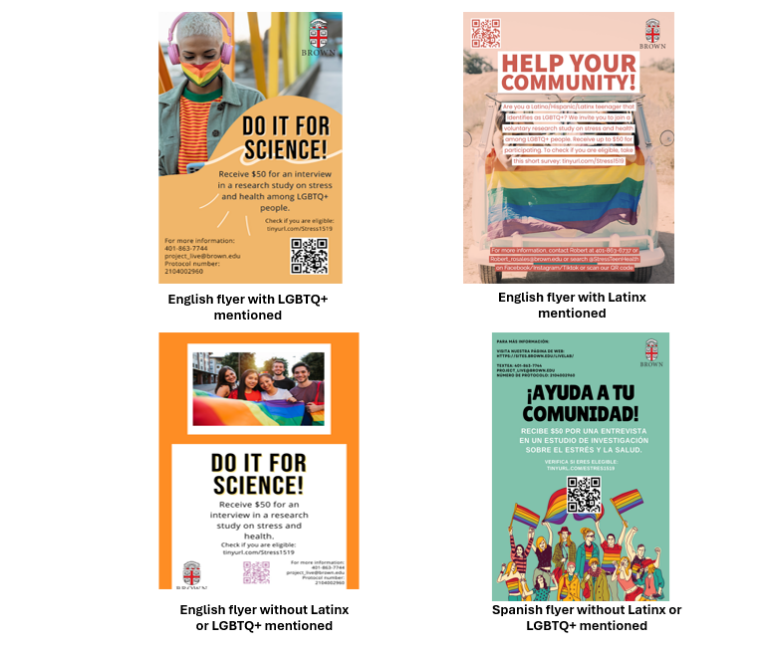
Examples of phase 1 study flyers. LGBTQ+: lesbian, gay, bisexual, transgender, and queer or questioning.

**Figure 2:**
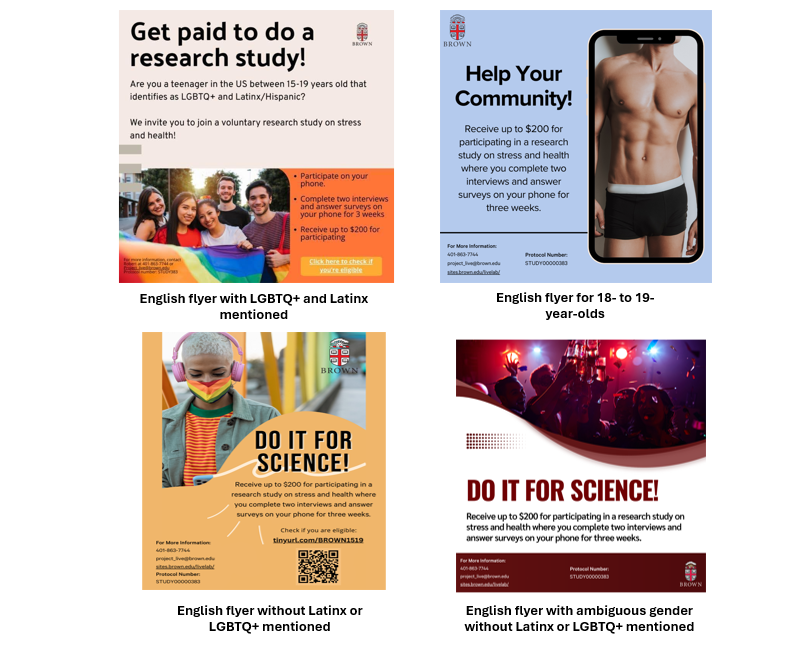
Examples of phase 2 and 3 study flyers. LGBTQ+: lesbian, gay, bisexual, transgender, and queer or questioning.

Multiple flyer versions were developed to reach distinct participant groups. Variations were based on recruitment modality. Flyers distributed in person were rectangular and included a QR code to direct participants to the eligibility screening survey. In contrast, digital flyers were square to meet Facebook and Instagram advertisement specifications; these did not include a QR code or link, as clicking the advertisement automatically redirected users to the screening survey. Flyers also varied according to the intended target population. For instance, materials aimed at Latinx SMY featured images of individuals visually identifiable as people of color and included the term “Latino/Hispanic/Latinx”. Additional variants included or excluded the term “LGBTQ+” (lesbian, gay, bisexual, transgender, and queer or questioning) to reach both youth who explicitly identify as LGBTQ+ and those who do not adopt such labels but report same-sex attractions or behaviors. Examples of flyers used in Phase 1 are presented in Figure 1, with additional examples from Phases 2 and 3 shown in Figure 2.

### Procedures

#### Screening Process

Interested individuals were redirected to an initial screening survey administered via Qualtrics. The screener assessed age, English/Spanish fluency, sexual identity, sexual behavior, sexual attraction, recent experiences with minority stressors, current alcohol use, ethnicity (Latinx vs non-Latinx White), race, and access to a phone or device with internet capability. The age item was revised from “Are you between 15 and 19 years of age?” (response categories: Yes/No) to “What is your age?” (open-ended response) after it was observed that some respondents older than 19 selected “Yes” to the original question to gain study access. The open-ended format was intended to reduce this misrepresentation by making eligibility criteria less transparent.

Participants were also asked to report their sex assigned at birth to facilitate a balance between those assigned male and those assigned female at birth. Beginning in Phases 2 and 3, an additional item was added to confirm access to an electronic device capable of supporting surveys and interviews. Finally, the screener included an item asking participants to identify where they saw or heard about the study, which provided information to refine and improve recruitment strategies. Screening questions and corresponding inclusion criteria are presented in Table 1.

**Table 1:** Qualtrics screening questions.

Inclusion criteria		Questions	Response categories	Inclusion response	
**Age**					
	Original	Are you between 15 and 19 years of age?	(1) Yes or (2) No	Yes	
	Revised	What is your age?	Open-ended	15-19 years old	
English or Spanish fluency		Do you speak fluent English or Spanish?	(1) Yes or (2) No	Yes	
Recently experienced discrimination		Have you been discriminated against in the last 30 days because of your sexual or racial identity?	(1) Yes or (2) No	Yes	
Current alcohol use		Have you had an alcoholic beverage in the last 30 days?	(1) Yes or (2) No	Yes	
**Race and ethnicity**					Either (1) Latinx and any racial category or (2) not Latinx and White
	Hispanic or Latinx	Do you identify as Hispanic/Latino/Latinx or were you or your parents born in Latin America?	(1) Yes or (2) No		
	Race	What is your Race?	(1) White, (2) Black/African American, (3) Asian, (4) Native Hawaiian or Other Pacific Islander, (5) American Indian or Alaska Native, and (6) Other		
**Dimensions of sexual orientation**					Only participants who respond in the following way are excluded (1) Straight/heterosexual (sexual minority identity), (2) Person of the same sex (sexual minority behavior), and (3) Person of the same sex (sexual minority attraction)
	Sexual minority identity	Do you identify as:	(1) Straight/heterosexual, (2) Gay, lesbian, bisexual, queer, (3) Other, (4) Not sure, and (5) Don’t know		
	Sexual minority behavior	During your life, with whom have you had sexual contact?	(1) I have never had sexual contact, (2) Person of the same sex, (3) Person of the opposite sex, and (4) Both sexes		
	Sexual minority attraction	Who are you sexually attracted to?	(1) I am not sexually attracted to anyone, (2) Person of the same sex, (3) Person of the opposite sex, and (4) Both sexes		
Ability to complete electronic surveys		Do you have a phone, computer, or device that you can use to respond to daily surveys and do video interviews?	(1) Yes or (2) No	Yes	
Recruitment location		Where did you find out about this study?	(1) Facebook, (2) TikTok, (3) Instagram, (4) Flyer at a clinic, (5) Flyer at a bus station, (6) Flyer at school, (7) A friend, (8) A teacher, (9) My clinician (example: social worker, psychologist), (10) Someone else (Please tell us who):, and (11) I saw a flyer somewhere else (Please tell us where):	N/A^a^	
Sex assigned at birth		What sex were you assigned at birth (on your original birth certificate)?	(1) Female, (2) Intersex, (3) Male, and (4) Prefer not to answer	Respondents assigned a certain sex at birth will be excluded or brought into the study when we are able to get a better balance of participants’ sex assigned at birth	

^a^Not applicable.

#### Challenges: Screening Out Bots

To further ensure that ineligible individuals did not enter the study, we implemented an additional screening interview. Initially, eligibility was determined solely through the Qualtrics screener. However, the first author observed indicators of ineligible participation. For example, some respondents appeared to be located outside of the United States, as suggested by discrepancies such as dark video backgrounds during morning hours in the United States and non-US time zones displayed in the Calendly scheduling system. In addition, as noted above, some participants self-reported ages exceeding the upper cutoff of 19 years.

Given such recruitment challenges, a structured screening interview was added to confirm eligibility. During this process, participants were asked to provide their zip code, state, birthdate, and age, and responses were cross-verified for consistency (eg, confirming that a reported zip code matched the reported state). The interviewer was authorized to determine eligibility by integrating all available data, including participant responses, time-of-day consistency, and indicators of potential fabrication (eg, evidence of online searching during questioning). We believe this procedure successfully excluded nearly all ineligible participants. Because a substantial number of screening interviews continued to involve ineligible respondents, we retained this additional verification step throughout the study.

Prior research indicates that open-ended questions, such as those asking about age, can be useful in detecting discrepancies that help identify bots and fraudulent participants [40,41]. Accordingly, we incorporated 4 additional items into the screening process to enhance data integrity: “What is your age?” “What is your birthday?” “Please share anything that will help prepare for our meeting” and “How did you hear about this study?” Responses to these items were reviewed and compared with the digital screener to verify consistency before scheduling the screening interview.

For further quality control, participant IP addresses were collected in Qualtrics. Cases were excluded when responses were inconsistent (eg, evidence of bot activity or mass-produced responses), when multiple entries were submitted by the same users, or when individuals appeared to be intentionally misrepresenting themselves (eg, attempting to qualify for an ineligible survey, providing contradictory information). In such instances, participants were not enrolled in the study, and compensation was withheld.

#### Phase 1: Qualitative Phase to Refine and Adapt Interview

Phase 1 consisted of cognitive interviews with approximately 20 SMY (half Latinx; half non-Latinx White) to refine and adapt survey items, reduce participant burden in the subsequent EMA survey, and ensure cultural and contextual appropriateness for SMY. Each interview lasted approximately 2 hours and was conducted via Zoom. The interview had 2 components. First, participants engaged in a think-aloud exercise, a method designed to capture participants’ real-time cognitive processes while completing survey items [42-44]. To familiarize participants with the approach, the session began with a practice question unrelated to the study (eg, “How many windows are there in your house, apartment, or dorm?”) [45]. Participants were then asked to verbalize their thoughts while completing the draft survey planned for Phases 2 and 3 [46]. These think-aloud responses were used iteratively to identify comprehension issues, detect errors, and refine survey items, consistent with best-practice recommendations [42]. Following the think-aloud portion, participants responded to a series of open-ended questions regarding (1) general feedback on survey items, (2) impressions of recruitment flyers, (3) alcohol use, (4) sociocultural risk and protective factors, (5) alcohol-related consequences, and (6) access to treatment.

#### Phase 2: Pilot Study With Follow-Up Interviews to Assess Survey Feasibility and Acceptability

During Phase 2, participants completed all study procedures remotely via Zoom. At the baseline session, participants were introduced to the study app on their personal mobile device and trained in the use of the EMA survey system. The baseline survey assessed demographics, covariates, and variables relevant to moderation analyses. At the beginning of the session, a trained research team member administered the Timeline Followback interview to capture alcohol and cannabis use over the previous 28 days [47]. Participants were then provided with a link to complete the remainder of the baseline survey. Surveys were completed while the research team member remained present to answer any questions or clarify item wording. Pilot testing indicated that the baseline interview required approximately 50 minutes to complete (range = 45 minutes to 1 hour). The Catalyst mobile app (MetricWire Inc), was used to administer EMA surveys in this study. Participants received brief training on how to download and install the app on their personal devices. Once installed, a study team member provided structured training on EMA procedures, including the types of prompts, survey frequency, procedures for problem reports, and options for participant-initiated reporting. Training also covered clarification of study terminology (eg, definitions of identity within the EMA survey, the meaning of a standard drink), using a live demonstration within the app.

Participants were asked to complete a final interview designed to inform refinements to the Phase 3 EMA survey methodology. During this interview, the Timeline Followback was re-administered to assess alcohol and cannabis use, allowing verification of whether any episodes had been missed in the EMA reporting. Participants also completed a mixed methods Qualtrics survey in which they provided feedback on the EMA survey, including any difficulties encountered or suggestions for improvement.

#### Phase 3: 21-Day EMA Protocol

Due to the pilot showing high feasibility and acceptability (manuscript expected in March 2026), the protocol for Phase 3 looks similar, with only changes to the final interview. Rather than reporting on the feasibility of the study, participants are asked to report on treatment adaptation needs, help-seeking, readiness to change, mobile health intervention preferences, and barriers to treatment. The aim of these questions is to provide preliminary findings on how to adapt an intervention for this population. In addition, because the feasibility and acceptability are high for this EMA protocol, all Phase 2 EMA responses have been combined with Phase 3 responses.

#### EMA Survey Structure

Participants engage in up to 21 consecutive days of EMA. Surveys assess alcohol and other substance use, sexual activity, mental health, and exposures to stressors (including discrimination). Consistent with prior EMA protocols assessing substance use [28,48], participants complete up to 6 compensated surveys per day: one morning survey, one bedtime survey, and 4 randomly prompted surveys (referred to as Teen Electronic Diary [TED] reports). In addition, participants have the option to initiate a problem report (to indicate technical or content-related issues) and a begin drink report (to mark the onset of alcohol consumption). These optional reports are not required and do not carry compensation.

**Figure 3:**
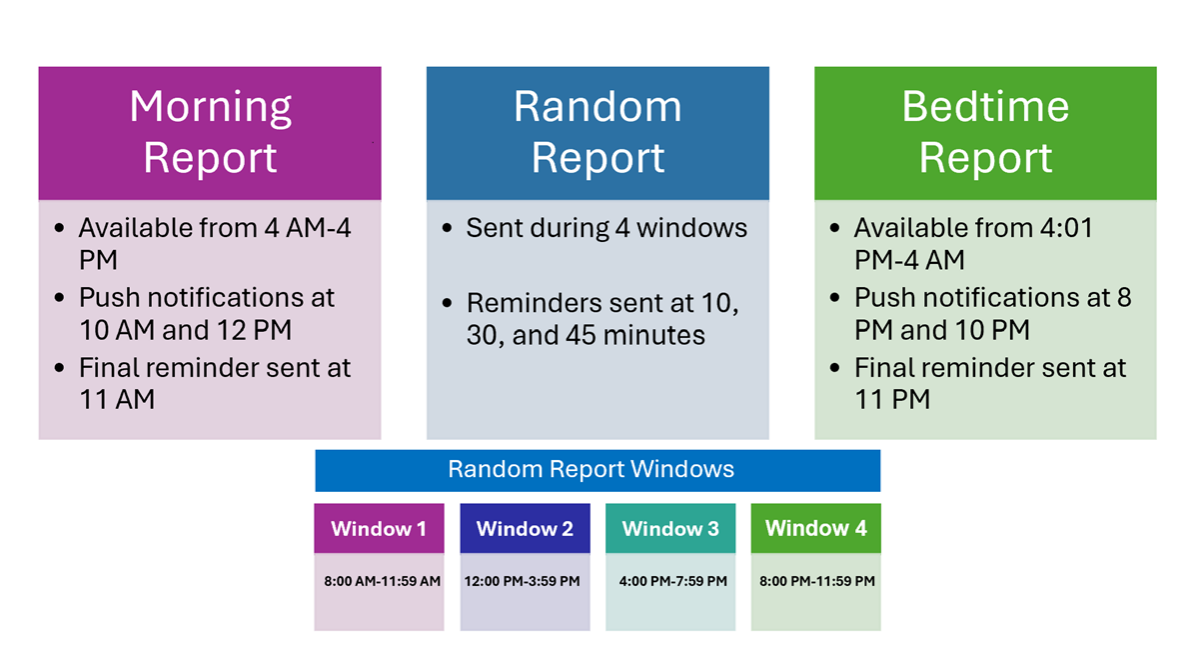
Description of morning, random, and bedtime report timing.

The EMA protocol includes 3 types of scheduled reports—morning, bedtime, and random—as well as optional self-initiated drinking reports. Figure 3 provides a description of the scheduling, prompts, and reminders for each type of these reports. Morning reports (ie, surveys completed by participants in the morning) assess sleep quality, unreported substance use from the previous day, and anticipated substance use. Bedtime reports assess social support and belongingness experienced during the day. Random reports (also called TED reports) assess real-time affect, substance use, and minority stress. Our random reports will be scheduled daily within 4 windows, and participants are instructed to complete each survey within 59 minutes of the first prompt. If a survey is still incomplete, SMS reminders will be sent (eg, “Hi, [participant First Name]. Please complete your TED Report ASAP. It will expire soon and your answers are important to us.”). Additional information about the timing of each report can be found in Figure 3. To minimize burden, participants are advised not to complete surveys in unsafe or inappropriate contexts (eg, while driving or during class). The final type of report, self-initiated drinking reports, allows participants to document the onset of alcohol use and associated experiences of minority stress in real time.

### Compensation

Participant compensation will vary by study phase but will be consistently delivered via the participants’ preferred e-gift card option. A detailed breakdown is presented in Table 2. In Phase 1, participants received US $50 for completing the 2-hour cognitive interview. In Phase 2, participants earned up to US $200 for full study participation. This included US $40 for the 1.5-hour baseline interview and US $25 for the 30-minute final interview. To encourage EMA compliance, participants were compensated per survey rather than through unannounced bonuses, a strategy shown to enhance adherence in EMA studies [49]. Specifically, participants earned US $1 per completed survey and an additional US $3 incentive for completing at least 75% of surveys within a 7-day period, for a potential EMA total of US $135. EMA compensation was disbursed weekly, with lump-sum payments issued each Monday after participants’ first 7 days in the study. Phase 3 compensation follows the same structure as Phase 2.

**Table 2:** Compensation breakdown.

Study activity		Compensation (US $)		Total possible compensation (US $)
**Phase 1**				50
	2-hour cognitive interview	50		
**Phase 2**				200
	First interview	40		
	21-day surveys on your phone	Up to 126 (1/survey)		
	Incentive for completing 75% of a week	Up to 9 (3/week)		
	Final interview	25		
**Phase 3**				175
	First interview	40		
	21-day surveys on your phone	Up to 126 (1/survey)		
	Incentive for completing 75% of a week	Up to 9 (3/week)		

### Measures

#### Phase 1: Qualitative Phase to Refine and Adapt Interview

During the item refinement portion of the qualitative interviews, participants responded to a battery of questions intended for use in the baseline and EMA portions of Phases 2 and 3. To align with the originally planned 30-day EMA period, all items were asked with a 30-day recall frame. First, participants completed the Everyday Identity Stress Scale (9 items), developed to assess daily experiences of identity-related stress among SMY [50] and subsequently adapted for momentary assessment in EMA studies [28]. Items included questions about distal discrimination (eg, “I was targeted or harassed because of my identity”) and attribution of experiences to specific identities (eg, “My race”). Second, participants completed the LGBTQ+ People of Color Microaggressions Scale (16 items), which measures 3 domains: LGBT racism (eg, “I felt misunderstood by White LGBTQ+ people”), People of Color heterosexism (eg, “I felt misunderstood by other Latino/Latinx/Hispanic people/person”), and LGBTQ+ relationship racism (eg, “I was rejected by potential dating or sexual partners because I am Latino/Hispanic”) [51]. Finally, participants were asked to respond to additional items identified as potential moderators or mediators, including victimization, social support, coping strategies, alcohol-related consequences, and drinking context. A complete list of items and response categories is provided in Table 3.

**Table 3:** Constructs measured in Phase 1 qualitative interviews.

Construct and questions				Response categories
**Victimization**				
	1) Were you bullied or harassed by someone in the last 30 days?		1) No 2) Yes	
	2) If yes, how did it happen: (Check all that apply)		1) in-person contact 2) by telephone 3) by text message 4) via online interaction 5) through some other way	
	3) Who bullied or harassed you? (Check all that apply)		1) Friends I drink with 2) Friends I don’t drink with 3) Classmates who are not friends 4) Someone online 5) Coworkers 6) Others	
**Social support**				
	1) After one of these negative experiences, someone expressed care/concern for me.		1) No 2) Yes	
	2) If yes, who? (Check all that apply)		1) Friends I drink with 2) Friends I don’t drink with 3) Classmates who are not friends 4) Boy/girlfriend/intimate partner 5) Child/children 6) Mother 7) Father 8) Siblings, brothers, or sisters 9) Other relatives 10) Friend’s parents 11) Coworkers 12) Others _________	
**Coping**				
	1) Did you use one or more of the following coping strategies to manage your emotions after experiencing discrimination in the last 30 days? (Check all that apply)		1) keep busy 2) socialize 3) think positively 4) do something good for self 5) calm self 6) find perspective 7) sit with feelings until they pass 8) use substances 9) get angry 10) ruminate 11) Other _____	
**Alcohol consequences**				
	1) Did you have any of the following problems in the last 30 days because of your drinking?		1) Legal problems (for example: related to citizenship) 2) School problems (for example: suspended or expelled from school) 3) Work problems (for example: being fired or decreased hours) 4) Relationship problems	
**Drinking context**				
	**Prompt: Think about the last time you drank alcohol. Answer the following questions as if you were in that situation.**			
		1) Where were you? (Check all that apply)	1) Home (a) 2) Dorm (b) 3) Friend’s home (c) 4) School (d) 5) Work (e) 6) At a party (f) 7) Restaurant (g) 8) Club or bar (h) 9) Outside in public place (i) 10) Inside in a public place (j) 11) Car, bus, other transportation (k) 12) Study visit (Brown) (l) 13) Elsewhere _______ (m)	
		2) Were you in a place where you usually drink?	1) No 2) Yes	
		3) Who was with you? (Check all that apply)	1) No one 2) Friends I drink with 3) Friends I don’t drink with 4) Family member 5) Friend’s family member 6) Coworkers 7) Others	
		4) How many people are with you?	Open ended	
		5) Were you with one of the following: (Check all that apply)	1) A Latino/Latinx/Hispanic person/people 2) An LGBTQ+^a^ person/people 3) An LGBTQ+ Latino/Latinx/Hispanic person/people	

^a^LGBTQ+: lesbian, gay, bisexual, transgender, and queer or questioning.

#### Phase 2 and 3 EMA Survey

**Figure 4:**
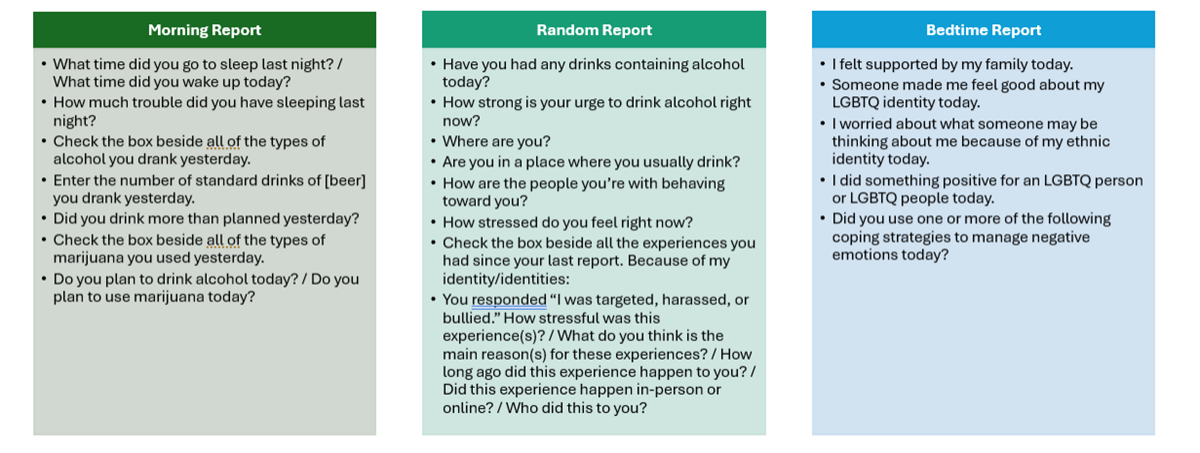
Examples of morning, random, and bedtime report questions. LGBTQ+: lesbian, gay, bisexual, transgender, and queer or questioning.

The EMA protocol incorporated morning, random, and bedtime reports to assess minority stress, protective factors, and ATOD use in real time. The morning and random report survey items were adapted from an EMA battery previously validated for alcohol use [52]. Random reports assessed momentary affect, substance use, cravings, situational context (eg, location, companions, drinking environment), and experiences of minority stress. The bedtime report questionnaire was adapted from prior research examining minority stress, affect, protective factors, and tobacco use among SMY [53]. This measure also incorporated items assessing ethnicity as a protective factor (eg, I was involved in an activity, meeting, event, or overall community for my ethnic group today). Bedtime reports captured participants’ daily experiences of minority stress and their responses to these experiences, including the use of coping strategies and the receipt of social support. Example questions for each report can be seen in Figure 4.

### Analytic Approach

#### Phase 1: Qualitative Phase to Refine and Adapt Interview Analytic Plan

For Phase 1, descriptive statistics were used to evaluate whether survey measures require refinement (see items in Table 3; manuscript expected in March 2026). In addition, thematic analysis was conducted using NVivo (QSR International) software to identify themes emerging from the qualitative interviews’ open-ended questions and to assess whether measures should be adapted using the think-aloud responses. Thematic analysis is a qualitative analysis technique for coding and deriving meaning from interview data [54]. Identified themes were connected to existing literature on minority stress and protective factors related to alcohol use. Based on these findings, the research team refined measures by incorporating items that reflect participants’ lived experiences (manuscript expected in March 2026). Participants were also asked to provide feedback on their experiences completing the survey. Follow-up questions included the following: (1) “How was your experience with filling out the questionnaire?” (2) “How much time do you think it took for you to complete the questionnaire? How do you feel about that?” (3) “How would you feel about taking it 3-5 times a day? For 30 days?” (4) “How much would you want to get paid if you had to do it this many times?” (5) “Were there any questions that you did not understand?” (6) “Were there any questions that were not related to your experience?” and (7) “Was there a question that you were expecting that was not there?”

#### Phase 2: Pilot Study With Follow-Up Interviews to Assess Survey Feasibility and Acceptability Analytic Plan

In Phase 2, qualitative data from the final interview following the 21-day EMA pilot will be thematically analyzed to identify themes related to the feasibility and acceptability of the EMA protocol (analyses planned for January 2026, and manuscript expected in March 2026). These themes will be integrated with EMA response rate data to evaluate potential modifications aimed at enhancing protocol adherence and improving the design of the finalized EMA survey in Phase 3.

#### Phase 3: 21-Day EMA Protocol Analytic Plan

Multilevel modeling (MLM) is the standard analytic approach for EMA data (manuscript expected March 2026 [52,55,56]. In Phase 3, MLM will be conducted in Mplus to examine the relationship between minority stress and alcohol use, minority stress and sociocultural risk and protective factors, sociocultural risk and protective factors and alcohol use, and whether these sociocultural risk and protective factors influence the association between minority stress and alcohol use. Four hypotheses will guide the analyses: (1) minority stress experienced in the moment will predict increases in SMYs’ alcohol use, (2) negative affect will mediate the association between minority stress and alcohol use, (3) ethnic identity (Latinx) will exacerbate the relationship between minority stress and alcohol use, and (4) protective factors (eg, social support, ethnic identity salience, and outness) will mitigate the relationship between momentary minority stress and alcohol use. The primary outcome of interest for phase 3 will be within-person alcohol use assessed in three separate ways: (1) in the moment, (2) the same day, and (3) on the weekends. Since we are not powered to assess between-person relationships, we also aimed to assess differences in between-person differences in these alcohol use rates.

All analyses will adjust for covariates that may function as potential confounders (ie, sex, age, and household income). A 2-level MLM will be estimated, in which survey responses (Level 1: L1) are nested within participants (Level 2: L2) [57]. L1 represents within-person effects (eg, momentary minority stress, affect, alcohol use), encompassing both event-level and random assessments and thus capturing within-subject variability. L2 represents between-person effects (eg, sex, ethnicity, or L1 variables aggregated across time), reflecting between-subject variability. To facilitate interpretation, L1 variables will be person-centered and L2 variables grand mean-centered. Compositional effects will be examined by aggregating the L1 variables to partition between-subject and within-subject variance. Minority stress responses from EMA surveys will be modeled as L1 predictors of the primary dependent variable, alcohol use. This approach will allow us to test whether slopes for alcohol use differ significantly between participants who experience minority stress and those who do not.

Moderators and mediators will be incorporated into the base multilevel model to test additional hypotheses. To evaluate hypothesis 2—that negative affect mediates the relationship between minority stress and alcohol use—we will include EMA reports of affective state (L1) as a mediator. This analysis will proceed in 2 stages (planned for February 2026). First, we will examine contemporaneous associations, testing whether minority stress (independent variable) is associated with alcohol use (dependent variable) and negative affect (mediator) at the same survey time point. This will assess whether stress and alcohol use co-occur within an event, as affect may be immediately influenced by stress experiences (or vice versa). Second, we will examine lagged associations, testing whether minority stress or negative affect at time *t* predicts alcohol use at Time *t*+1 (ie, the subsequent EMA prompt, several hours later). This temporal ordering provides a stronger test of mediation by assessing whether earlier stress or affect predicts later alcohol use.

We will also estimate a lagged model to examine whether minority stress predicts alcohol use on the following day or weekend. Formal tests of multilevel mediation will be conducted using the procedures outlined by Zhang, Zyphur, and Preacher [58]. Given the relatively modest sample size, emphasis will be placed on effect sizes rather than significance tests when interpreting mediated effects. Consistent with hypothesis 3, ethnicity (White vs Latinx) will be included as a moderator in the base model to assess support for either the double jeopardy or resilience hypothesis. Specifically, we hypothesize that Latinx SMY who experience discrimination (double jeopardy) will report higher alcohol use relative to White SMY. Finally, additional moderators will be examined to evaluate protective influences on the minority stress-alcohol use relationship. These will include baseline characteristics (eg, coping strategies, ethnic identity, level of outness, and gender) and daily-level variables (eg, social support and belongingness).

## Results

### Overview

In this section, we will present descriptive findings on sample characteristics, recruitment sources, feedback from the Youth Advisory Board (YAB), and data from our digital recruitment platform (Meta Business). Together, these results provide insights into the barriers and facilitators of conducting research with this population and may inform the design of future studies.

### Sample Characteristics

Phase 1 was completed with 23 participants, recruited between April 2022 and November 2023. Descriptive statistics for the sample are presented in Table 4. Thirteen (57%) participants identified as Latinx and 10 as non-Latinx White, which met our original goal of dividing participant ethnicity in half. The average age was 18.1 years, and the mean reported household income was US $112,449.50. A majority of participants were enrolled in college (17/23, 74%) rather than high school. Although most participants were assigned female sex at birth (18/23, 78%), the sample reflected considerable diversity in gender identity. Specifically, participants self-identified as gender fluid (n=1), gender queer (n=5), men (n=4), non-binary (n=5), transmen (n=5), and women (n=10).

We assessed both sexual identity and sexual attraction as dimensions of participants’ sexual orientation. Regarding sexual identity, the largest proportion of participants (9/23, 39%) identified as bisexual. With respect to sexual attraction, the largest group (10/23, 44%) reported attraction to people of any or all genders.

Participants were also asked to report their national background. The majority (19/23, 82%) were born in the United States. Latinx participants were additionally asked to specify their national heritage. Among this subgroup, the largest proportions identified as Dominican (3/13, 23%) and Colombian (3/13, 23%).

**Table 4:** Phase 1 participant demographics.

Construct		Values
**Ethnicity, n (%)**		
	Latinx	13 (57)
	Non-Latinx White	10 (43)
**Current education, n (%)**		
	In high school	6 (26)
	In college	17 (74)
**Sex assigned at birth, n (%)**		
	Female	18 (78)
	Male	5 (22)
**Gender identity^a^, n (%)**		
	Gender fluid	1 (4)
	Gender queer	5 (22)
	Man	4 (17)
	Non-binary	5 (22)
	Transman	2 (9)
	Woman	10 (44)
**Sexual orientation^a^, n (%)**		
	Asexual	1 (4)
	Bisexual	9 (39)
	Gay	4 (17)
	Lesbian	5 (22)
	Pansexual	3 (13)
	Queer	1 (4)
	Not listed	1 (4)
**Sexual attraction^a^, n (%)**		
	Both men and women	5 (22)
	Men	4 (17)
	People of any/all gender	10 (44)
	Women	5 (22)
	Non-binary people	1 (4)
**Nativity, n (%)**		
	US born	19 (82)
	Dominican Republic	2 (9)
	Colombian	2 (9)
**National heritage^a,b^, n (%)**		
	Mexican	1 (8)
	Dominican	3 (23)
	Central American	1 (8)
	Colombian	3 (23)
	Cuban	1 (8)
	Brazilian	1 (8)
	Other	1 (8)
	Not reported	2 (16)
Age (years), mean (SD)		18.13 (1.29)
Household income, mean (SD)		112,449.50 (88,860.91)

^a^Numbers may exceed 23 because participants were allowed to report more than one response.

^b^Only asked to the Latinx participants.

### Recruitment

#### Phase 1: Participant Location

During Phase 1, recruitment efforts reached (ie, number of participants who completed the digital screener) 896 potential participants. The majority were referred through Instagram (448/896, 50%), followed by Facebook (143/896, 16%), school-based flyers (81/896, 9%), friends (72/896, 8%), and other sources such as family, college email announcements, or community organizations (63/896, 7%). Due to confidentiality constraints, it was not possible to directly link screening questionnaires with the participants who ultimately enrolled in the study.

#### Youth Advisory Board Feedback

We also consulted Dr. Jackson’s YAB on 2 occasions to gather feedback on recruitment strategies and flyer design. The YAB consisted of racially diverse, primarily female high school students aged 15-18 years. Members emphasized the importance of visually appealing recruitment materials, noting a preference for flyers with vibrant colors and professional design. Trust emerged as a central theme in their feedback. Specifically, they advised that the inclusion of the Brown University logo and a study email with a Brown University domain increased their confidence that the study was legitimate. Conversely, the use of a tinyurl link to the study screening survey reduced trust, as it did not reflect a university-affiliated domain. Although the tinyurl was ultimately used because it was shorter and easier to type than the original Qualtrics link, future studies may consider adopting university-branded short links to strengthen perceptions of credibility and trust among potential participants.

#### Meta Business Suite Flyer Data

An iterative process was used to evaluate and refine flyer effectiveness using analytics from Meta’s Business Suite. This platform provides metrics, including results (link clicks), reach (number of unique individuals who viewed the advertisement), impressions (total number of times the advertisement was displayed), cost per click, and total spending. For example, between October 3 and October 10, one campaign generated 60 link clicks, reached 5922 individuals, and accumulated 10,407 impressions. The average cost per link click was US $0.73, with a total expenditure of US $43.52. To assess performance, we categorized advertisements with a cost per click below US $0.30 as high-performing, from US $0.30-US $0.40 as average-performing, and above US $0.40 as poor-performing. Advertisements exceeding US $0.40 per click were automatically replaced with alternative flyers. Initially, flyer performance was evaluated over a 7-day review period. However, this timeframe was extended to 30 days, as one week was insufficient to accurately assess campaign performance. Only a small number of flyers achieved high-performance status (ie, <US $0.30 per click). The strongest performer to date was the English language flyer for 18- to 19-year-olds (Figure 2), which consistently achieved a cost of approximately US $0.09 per click. We speculate that the flyer’s imagery, which featured a more sensual depiction of male bodies, may have contributed to its effectiveness in attracting participants.

Findings from the initial 7-day performance reviews also informed decisions about which flyer to use for in-person recruitment. Flyers that demonstrated the greatest efficiency in attracting participants online were prioritized for physical distribution. For example, Flyer 1 (Figure 1) performed strongly in digital recruitment and received high ratings from the YAB. As a result, this flyer was frequently used in in-person recruitment, with minor modifications to tailor messaging to specific communities. For instance, flyers disseminated in Latinx neighborhoods included explicit references to Latinx/Hispanic identity to better align with the target audience.

#### Phase 2 and 3: Recruitment Status

Phase 2 recruitment started in May 2024 and ended in November 2024. Phase 3 began immediately after Phase 2 and has a projected end date of February 2026. As of the initial submission of this manuscript (November 6, 2025), 10,197 people have completed the screening survey, and 355 of them have scheduled a screening interview. From this, 260 participants were excluded (ie, not eligible [n=23], no-shows [n=20], suspected to be fraudulent [n=72], and other reasons [n=145]), and 95 participants were deemed eligible. Of the 95 eligible participants, 79 participants completed the baseline session and EMA protocol (15 participants were lost to contact, and one participant was no longer interested). Fifty-three participants have completed the final interview (with 4 participants still actively completing EMA). Data has been cleaned for 54 participants.

### Challenges Confronted

**Figure 5:**
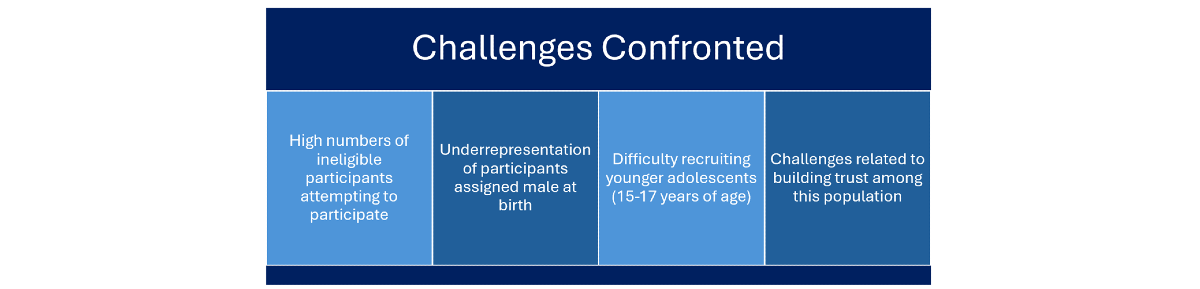
Challenges encountered during the study.

The primary challenges encountered in this study centered on recruitment (Figure 5). Specifically, we faced difficulties with ineligible participants attempting to enter the study, underrepresentation of participants assigned male at birth, limited recruitment of younger adolescents, and the need to build trust within the community. Similar challenges have been documented in other research with racial/ethnic and sexual minority populations. In particular, the shift toward digital research during and after the COVID-19 pandemic has been associated with increased instances of participants misrepresenting their demographic information, as well as automated “bots” attempting to complete screening surveys, issues that disproportionately affect studies involving underrepresented groups [40,59]. Our experience demonstrated that a web-based screener alone was insufficient to exclude ineligible respondents. To address this, we implemented additional safeguards, including digital screening interviews, open-ended questions, the collection of general location information, and cross-verification of responses to detect discrepancies. These procedures substantially reduced the likelihood of ineligible participants entering the study.

Second, consistent with other research [60], we encountered challenges in recruiting participants assigned male at birth. Despite initially implementing strategies intended to enhance male participation (eg, waiving parental consent) [61], recruitment of male participants—particularly during Phase 1—remained limited. We observed some improvement later by tailoring recruitment materials specifically for this group, including flyers and campaigns that featured male-presenting individuals and people of color in the visuals, particularly to engage Latinx SMY boys and young men [62]. Although this approach increased male participation, enrollment levels did not reach parity with female participants.

Thirdly, we faced challenges in recruiting adolescents aged 15-17 years during Phase 1. To increase enrollment among this age group, we implemented several strategies: (1) incorporating age-appropriate imagery into flyers, (2) expanding outreach and advertising efforts within high schools, and (3) launching a Facebook advertisement campaign, specifically targeting youth aged 15-17 years. These strategies yielded modest improvements, though recruitment in this age group remained more difficult than among older participants.

Finally, we confronted challenges related to building trust among this population, which may have limited participation among lower-income youth and 17-19-year-old youth not enrolled in college. The Phase 1 sample was more affluent and more highly educated than the general US population. One possible explanation is that the use of the Brown University logo enhanced credibility among college-attending and affluent youth, while offering little name recognition in lower-income communities. In some cases, institutional branding may even deter participation if potential participants fear being exploited by researchers. Partnering with nationally or locally recognized community organizations (eg, The Trevor Project at the national level or Youth Pride Inc. of Rhode Island locally) may therefore represent a more effective strategy for fostering trust and engagement among underrepresented subgroups in future research.

## Discussion

### Principal Findings

This paper describes the protocol and lessons learned from a 3-phase mixed methods study examining minority stress, risk and protective factors, and ATOD use in real time among Latinx and non-Latinx White SMY. More specifically, we predicted the following: in-the-moment minority stress will predict increases in SMY alcohol use; negative affect will mediate the association between minority stress and alcohol use; Latinx identity will exacerbate the relationship between minority stress and alcohol use; and protective factors will mitigate this relationship. The roadmap developed through this work has enabled the recruitment and enrollment of 101 participants as of November 2025 and provides a practical framework for future researchers seeking to recruit, verify eligibility, administer surveys and qualitative interviews, or implement EMA protocols with SMY populations. Elements of this roadmap may be adapted or adopted in whole or in part to enhance the efficiency of similar studies. We highlight examples of effective recruitment flyers and strategies, including benchmarks used to assess campaign performance. We also present insights from our YAB, whose feedback improved campaign effectiveness by shaping flyer appropriateness, design, and perceived trustworthiness. Importantly, this roadmap reflects nearly 5 years of iterative research and troubleshooting during which numerous challenges were encountered and addressed.

### Building Off Prior Work

This study protocol builds on prior seminal work using EMA to assess the effects of minority stress on behavioral health. Our EMA protocol and survey were modeled after the study by Mereish et al [28] assessing the effects of minority stressors on nicotine use among SMY, which used a similar method (eg, inclusion criteria, EMA battery, and incentive structure). This study found that (1) minority stressors were associated with greater momentary nicotine craving and negative affect, and (2) nicotine craving and positive affect mediated the relationship between minority stressors and nicotine use [28]. We also built off the EMA study by Livingston et al [14] with sexual minority adults, which showed that minority stressors in the moment were associated with greater odds of momentary nicotine and substance use. These studies have all been informed by the Psychological Mediation Framework, which states that emotion regulation (eg, negative affect) mediates the relationship between distal stigma-related stressors (eg, SMY discrimination) and psychopathology (eg, substance use) [63]. Our study goes beyond this prior research to assess whether ethnicity and the social oppressive structures that ethnic minority people experience differentiate the experiences between Hispanic and non-Hispanic White SMY.

### Strengths and Limitations

This study is not without limitations. First, our study is not fully powered to assess between-person differences in the effects of predictors on alcohol use. However, it may provide signals suggesting statistically significant relationships for a future fully powered EMA survey. Second, our samples of interest may be heterogeneous due to differences by country of origin, sexual orientation, and other factors related to ethnicity and sexual minority status. Findings for this study will be presented as general to the youth who identify as Hispanic and sexual minority or sexual minority. The findings will inform general experiences of these populations, and we will advocate for a larger study that can disentangle the heterogeneous experiences and studies on specific groups who may be at greatest risk for alcohol use (eg, Puerto Rican SMY). Third, our study has primarily enrolled participants who are in college, upper-middle-income, and assigned female at birth. Future studies should use other recruitment techniques to recruit greater numbers of lower-income and male participants. One such technique, which was not approved by the Brown University IRB, could involve using respondent-driven sampling to compensate low-income, non-college-educated, and male participants to recruit similar participants in their community. Respondent-driven sampling has been used to recruit large samples of participants from marginalized communities into similar research studies within weeks [64,65].

### Future Directions

The lessons learned from this study provide a foundation for several ongoing and planned projects within our research group. These include manuscripts examining (1) differences in minority stress and alcohol use between Latinx SMY and non-Latinx SMY using Phase 1 qualitative interviews, (2) feasibility and acceptability of the EMA protocol from Phase 2, and (3) risk and protective factors associated with momentary alcohol and cannabis use among SMY. Beyond informing our own work, we hope this paper will serve as a roadmap for other researchers working with this population. More broadly, our aspiration is to contribute to the development of best practices for conducting research with intersectionally marginalized youth, including those who are both sexual minority and racial/ethnic minority.

### Dissemination Plan

In addition to submitting the above-mentioned manuscripts to peer-reviewed academic journals, we plan to disseminate the findings of these studies to lay audiences. We have already used some of the lessons learned about behavioral health in this population to write an op-ed in the Miami Herald calling for the repeal of the colloquially termed “Don’t Say Gay” and “Stop Woke” acts [66]. We plan to submit similar op-eds using the findings from this study to support policy change for Hispanic and non-Hispanic SMY. In addition, we plan to write policy briefs that can be presented to policymakers at policy summits, in reports, and to support anti-oppressive legislation. Finally, we aim to have these findings readily available to parents of SMY. Thus, we plan to share our findings with community organizations and parenting groups with the aim of providing caregivers with skills on how to best support youth in this community. We also plan to submit our findings to media targeting this caregiver population, such as Spanish news media (eg, La Opinión) and major news media with articles focused on supporting this population (eg, ABC News’ Family section).

## Appendix

Multimedia Appendix 1 Summary Statement from NIH Reviewers.

## Abbreviations

ATOD: alcohol, tobacco, and other drugs
EMA: ecological momentary assessment
IRB: Institutional Review Board
L1: Level 1
L2: Level 2
LGBTQ+: lesbian, gay, bisexual, transgender, and queer or questioning
MLM: multilevel modeling
MST: minority stress theory
SMY: sexual minority youth
TED: Teen Electronic Diary
YAB: Youth Advisory Board

## Data Availability

The datasets generated or analyzed during this study are available from the corresponding author on reasonable request.
