# Supplementary material for: Real-Time Exposure to Intersectional Minority Stressors and Alcohol Use: Protocol for an Ecological Momentary Assessment Study With Latinx and Non-Latinx Sexual Minority Youth
Source: JMIR Res Protoc. 2026 Jan 30;15:e87201. doi: 10.2196/87201 (PMC12905568; doi:10.2196/87201)
Supplement: Multimedia Appendix 1 [file resprot_v15i1e87201_app1.pdf]

SUMMARY STATEMENT
(Privileged Communication)

PROGRAM CONTACT:
Nadra Tyus
301.402.1366
nadra.tyus@nih.gov

Release Date: **11/18/2020**
Revised Date: **11/18/2020**

Application Number: **1 K08 MD015289-01A1**
Formerly: **1K08MD015289-01**

Principal Investigator

ROSALES, ROBERT

Applicant Organization: BROWN UNIVERSITY

Review Group:	ZMD1 XLN (J1)<br>National Institute on Minority Health and Health Disparities Special Emphasis Panel<br>NIMHD Research Career Development Awards (Ks)

Meeting Date:	11/02/2020	*RFA/PA:*	PA20-203
Council:	JAN 2021	*PCC:*	IBB03NT
Requested Start:	04/01/2021	*Dual PCC:*	EB/KEA
		Dual IC(s):	DA

Project Title:	Socio-Cultural Protective and Risk Factors of Alcohol Use among Non-Hispanic White and Hispanic Sexual Minority Youth
SRG Action:	Impact Score:35
Next Steps:	Visit https://grants.nih.gov/grants/next_steps.htm
Human Subjects:	30-Human subjects involved - Certified, no SRG concerns
Animal Subjects:	10-No live vertebrate animals involved for competing appl.
Gender:	1A-Both genders, scientifically acceptable
Minority:	1A-Minorities and non-minorities, scientifically acceptable
Age:	6A-Children and Adults, scientifically acceptable

Project Year	Direct Costs Requested	Estimated Total Cost
1	153,080	165,326
2	156,922	169,476
3	160,880	173,750
4	164,956	178,152
5	169,155	182,687
TOTAL	804,993	869,392

ADMINISTRATIVE BUDGET NOTE: The budget shown is the requested budget and has not been adjusted to reflect any recommendations made by reviewers. If an award is planned, the costs will be calculated by Institute grants management staff based on the recommendations outlined below in the COMMITTEE BUDGET RECOMMENDATIONS section.

1K08MD015289-01A1 Rosales, Robert

RESUME AND SUMMARY OF DISCUSSION: This is a resubmission application submitted in response to PA20-203, Mentored Clinical Scientist Research Career Development Award (Parent K08 - Independent Clinical Trial Not Allowed). The purpose of the project is to provide insight on the effects of minority stress, affect, and sociocultural protective factors on Hispanic and White sexual minority youth's (SMY) use of alcohol. The Candidate is an excellent postdoctoral fellow and has strong academic training with bilingual social work background in mental health and alcohol use. Since the initial review, the Candidate has published three additional papers and submitted five additional manuscripts, demonstrating high productivity. The proposed training plan is well-articulated and includes a balanced mix of coursework, seminars, and meetings, which is clearly related to the research objectives for achieving research independence. The proposed research plan had several strengths including the high significance of this research topic given the high rates of alcohol use among SMY, the innovative ecological momentary assessment (EMA) approach and the mixed-method design, the rooted theory with clearly articulated hypotheses, and a balance of rigor and minimal participant burden. The mentoring team is strong with substantial, complementary expertise and an excellent track record of mentoring and training junior faculty. The environment is outstanding and supportive of the applicant with an Alcohol and Addiction Studies Center. However, some Panel Members noted that the Candidate has limited experience and publications related to sexual gender minorities. This resubmission was considered moderately responsive to prior review. A number of minor to moderate weaknesses around the research plan were still noted, and the primary concerns were related to the rigor of prior research, the overall coherence of research strategies, the sample size for Aim 3, the heterogeneity of the underdefined Hispanic and White SMY, the rationale for some hypotheses, the testing plan of mediation and moderation that are not well-detailed and in some ways are inaccurately used in the context. Overall, this application from an excellent candidate focused on an important topic is rated in the range of Good to Outstanding and could have medium impact.

DESCRIPTION (provided by applicant): NIAAA identifies underage drinking as a public health issue with serious consequences. Sexual minority youth (SMY) who use alcohol have even worse consequences, such as increased risk of cancer, risky sexual behavior, and suicidal attempts, due to their stigmatized identity. Hispanic SMY may be at even greater risk of developing alcohol use problems because of the additive status-based discrimination that may arise from being a sexual and ethnoracial minority. Minority stress theory posits that youth who experience discriminatory events cope with psychological distress by using alcohol. To date, little is known about how Hispanic SMY respond to social stressors or how socio-cultural protective factors (i.e., coping strategies, social support) and negative and positive affect may mediate these stressors' effects on Hispanic SMY's use of alcohol. EMA is a unique method that can assess minority stress theory, psychological distress, and alcohol use in real-time, however it has not been used to assess this relationship with Hispanic SMY. In line with NIH's recent call for increased research on sexual minority health, the research aims of this Career Development Award (K08) are to: 1) Assess the acceptability and feasibility of the minority stress, alcohol, and protective factor measures in ecological momentary assessment (EMA) between Hispanic and white SMY; 2) Examine the effects of minority stress on SMY's alcohol use and the mediating effects of affect; and 3) Examine the sociocultural risk and protective factors of alcohol use. This study will be conducted in 3 phases. In Phase 1, the measures will be developed using cognitive interviews with Hispanic and white SMY (N=20 or until saturation is reached). Phase 2 will consist of a pilot-EMA testing of the EMA with Hispanic and white SMY (N=20) over two-weeks and post-EMA cognitive interviews to assess the feasibility and acceptability of the EMA. In phase 3, the finalized EMA will be conducted over a 30-day period to assess minority stress, affect, and sociocultural protective factors of alcohol use among 50 Hispanic and 50 white SMY. This K08 proposed study and mentor plan will help the candidate develop expertise in 1) The development and progression of alcohol use in youth; 2) Risk and protective factors of alcohol use in SMY; 3) Ecological Momentary Assessment (EMA) methodologies; 4) Intensive longitudinal data analysis for analyzing EMA; and 5) cultural adaptation of

measures and interventions for SMY of color. The training goals will build off of the candidate's prior training in 1) Positive youth development among youth of color; 2) The effects of discrimination on substance use; 3) qualitative analysis, and 4) advanced statistical analysis.

PUBLIC HEALTH RELEVANCE
The present study aims to provide insight on the effects of minority stress, affect, and sociocultural protective factors on Hispanic and white sexual minority youth's (SMY) use of alcohol. The study will test whether the double jeopardy or resiliency hypothesis predict use of alcohol and the sociocultural protective factors that explain the differences between Hispanic and white SMY alcohol use. Findings will help to identify risk of alcohol use among SMY and the protective factors that could be leveraged in treatment to help address problematic drinking.

CRITIQUES: The written critiques of individual reviewers are provided in essentially unedited form below. These critiques were prepared prior to the meeting and may not have been revised afterwards. The "Resume and Summary of Discussion" above summarizes the final opinions of the committee.

CRITIQUE 1

Candidate: 2
Career Development Plan/Career Goals /Plan to Provide Mentoring: 1
Research Plan: 5
Mentor(s), Co-Mentor(s), Consultant(s), Collaborator(s): 3
Environment Commitment to the Candidate: 3

Overall Impact

This resubmitted K08 application focuses on elucidating risk and protective factors for alcohol use in Hispanic sexual minority adolescents. The topic is important given the high rates of alcohol use among sexual minority and little research on Hispanic sexual minority use. The use of ecological momentary assessment (EMA) to study the questions in this population is innovative and the mixed-method design is a strength. The candidate has strong background in mental health among Hispanics and research methods. He has also been quite productive since the original grant submission. The career development plan is strong, with clear relevance to the research plan and good balance between didactics, mentoring, and research outputs. The mentoring team has substantial expertise in all key aspects of the proposal, including alcohol use, EMA methods, mixed methods, Hispanic immigrants, and sexual minority youth. The research environment is excellent for the proposed training and research plans. The resubmission was moderately responsive to prior review, with multiple major weaknesses in the research strategy still present. These include confusion between mediation and moderation, lack of rationale for some hypotheses, and concerns about testing of mediation and moderation. These weaknesses diminish the potential impact of the application to a moderate level.

1. Candidate

Strengths

- Candidate received PhD in social work from Boston College in 2018.
- Has strong clinical and research experiences working with Hispanic clients as a social worker and studying Hispanics' access to mental health care.
- Solid training and experience with both quantitative and qualitative methods.
- T32 fellowship at Brown Center for Alcohol and Addiction Studies since 2018 focusing on etiology and treatment of alcohol abuse in Hispanics, experience with RCTs.

- Candidate has 13 published papers (5 as first author) and submitted 4 additional first-authored manuscripts on substance use among Hispanics since the previous submission.

Weaknesses

- None.

2. Career Development Plan/Career Goals & Objectives

Strengths

- Training goals include developing expertise in youth alcohol use, risk and protective factors for SM youth alcohol use, use of EMA, intensive longitudinal data analysis, methods of cultural adaptation for measures and interventions for SMY youth of color.
- Multiple collaborative opportunities on mentors' research projects.
- Research group meetings and seminars, directed readings, and coursework on sexual minority (SM), Intensive Longitudinal Data Methods, Multilevel modeling, Causal Mediation Analysis, IRT.
- Plans to submit three manuscripts (2 first-authored) each year, specific topics listed, and an R34 grant to develop an intervention based on K08 findings.
- Clear plan for frequency and activities during mentoring meetings.
- Good balance between training components.

Weaknesses

- None.

3. Research Plan

Strengths

- Sexual minority youth (SMY) experience much higher rates of alcohol use than their peers.
- Ethnic minority SMY experience more discrimination and rejection, but may also possess unique protective factors; the roles of these risks and specific protective factors have not been studied among Hispanic SMY.
- Generally strong rigor of prior research, with improved grounding of study hypotheses in relevant theoretical and empirical literatures.
- Use of EMA to study minority stress and alcohol use in Hispanic SMY is innovative.
- Qualitative pilot studies informing the main quantitative study; sample sizes were increased from 10 to 20 youth in each pilot study which should lead to more generalizable results
- Substantial sample size of 100 for the EMA study; 30 days should provide sufficient daily data; study well powered to detect small effects; within and between subject effect of time-varying predictors will be separated, lagged effects are included.
- Appropriate inclusion criteria for study aims and to ensure variability in discrimination and alcohol use.

Weaknesses

- No definition of SMY in Aims or Research Strategy; heterogeneity among different types of SM not addressed.

- If some youth respond to minority stress with greater substance use but others respond by developing resilience and lower substance use as hypothesized in Significance, then variable-centered analyses are not a good fit to test these hypotheses; instead, person-centered analytic approaches would be more appropriate.
- Conceptual confusion between mediation and moderation continues to be a major weakness; some mediation/moderation studies are not described correctly; the propositions of positive affect, social support, and coping as mediators are not supported by any theory or empirical research, and in the case of positive affect they contradict the minority stress theory that forms the theoretical basis of the proposal.
- Unclear why barriers to completion will only be discussed with participants who completed 0 time points in Phase 2, but not with participants who had low completion rates greater than 0; this is a missed opportunity to improve adherence in Phase 3.
- Mediation tests should include lags between predictor and mediator, as well as mediator and outcome, as recommended by relevant methodological literature.
- No description of how moderation will be tested; confusion between moderation and mediation in tests of Hypothesis 3.

4. Mentor(s), Co-Mentor(s), Consultant(s), Collaborator(s)

Strengths

- Mentor, Dr. Lee, has expertise in alcohol use among Latinx populations, immigrant stress, and mixed methods.
- Co-mentor, Dr. Colby, is a productive investigator with expertise in adolescent substance use and EMA studies.
- Co-mentor, Dr. Jackson, has expertise in longitudinal designs and analyses of substance use in youth.
- Co-mentor, Dr. Miranda, has expertise in using EMA to study minority stress in SMY tobacco use.
- Co-mentor, Dr. van der Berg, adds expertise in SMY.
- Mentor letters are enthusiastic and specific.

Weaknesses

- Candidate already worked with primary mentor, Dr. Lee, and co-mentor, Dr. Colby, during postdoc.
- Primary mentor has limited publication record.

5. Environment and Institutional Commitment to the Candidate

Strengths

- Center for Alcohol and Addiction Studies at Brown University has excellent resources and infrastructure for research and junior investigators.
- Center director and department chair express support for the candidate.

Weaknesses

- Institutional commitment letter states that the candidate’s faculty position will be “based on existing external funding”, although it specifies that it will not be dependent on whether this K application is funded; these statements seem to conflict.

Protections for Human Subjects

Acceptable Risks and Adequate Protections

- Risks are minimal, adequate protections are in place.

Data and Safety Monitoring Plan (Applicable for Clinical Trials Only):

Not Applicable (No Clinical Trials)

Inclusion Plans

- Sex/Gender: Distribution justified scientifically
- Race/Ethnicity: Distribution justified scientifically
- For NIH-Defined Phase III trials, Plans for valid design and analysis: Not applicable
- Inclusion/Exclusion Based on Age: Distribution justified scientifically
- Focus is on adolescents 15-19 years old, 50% females, 50% Hispanic and 50% White.

Vertebrate Animals

Not Applicable (No Vertebrate Animals)

Biohazards

Not Applicable (No Biohazards)

Training in the Responsible Conduct of Research

Acceptable

Comments on Format (Required):

- Online and meetings.

Comments on Subject Matter (Required):

- Key topics covered.

Comments on Faculty Participation (Required; not applicable for mid- and senior-career awards):

- Mentors will discuss ethical issues.

Comments on Duration (Required):

- Throughout grant duration.

Comments on Frequency (Required):

- Weekly.

Select Agents

Not Applicable (No Select Agents)

Resource Sharing Plans

Not Applicable (No Relevant Resources)

Authentication of Key Biological and/or Chemical Resources

Not Applicable (No Relevant Resources)

Budget and Period of Support

Recommend as Requested

CRITIQUE 2

Candidate: 1
Career Development Plan/Career Goals /Plan to Provide Mentoring: 1
Research Plan: 3
Mentor(s), Co-Mentor(s), Consultant(s), Collaborator(s): 3
Environment Commitment to the Candidate: 2

Overall Impact

This is a well written and well considered resubmission of a K08 from a very well qualified and promising Hispanic and bilingual candidate. Training plan is very well articulated and well thought out to fill specific gaps important in candidate's progression to independence including causal mediation analysis. Mentoring team is very strong and co-mentors have significant experience with fostering the careers of junior investigators. Research is rooted in theory with clearly articulated hypotheses and a balance of rigor and minimal participant burden. Environment is strong with an alcohol-focused research center. Mentoring background of primary mentor is not described and sample size for Aim 3 seems small given the number of variables assessed.

1. Candidate

Strengths

- Candidate is a Hispanic, bilingual social worker who has a nice portfolio of prior clinical and research experience in alcohol disparities and strong academic training.
- Since initial submission, candidate has published 3 additional manuscripts and submitted five additional ones.

Weaknesses

- Has limited SMY experience but mentorship team is experienced.

2. Career Development Plan/Career Goals & Objectives

Strengths

- Training plan's focus on learning more about alcohol use among youth, sexual minorities, methods of cultural adaptation, as well as EMA and longitudinal data analysis will build nicely on prior experience and linkage to long-term goals nicely explicated. Well-delineated sets of hands on learning experiences and course work per year.

- Details of individual meetings with mentors and advisors now presented as well as specific areas of trainings by mentor and co-mentors delineated.
- Plan now includes specific manuscript goals to increase experience and productivity.
- Development plans include submission of a K23 and very well-articulated plan to learn about and discuss ethical concerns related to very sensitive topic amongst a vulnerable population.

Weaknesses

- None significantly noted.

3. Research Plan

Strengths

- Strong integration of theory and clearly presented theory-based hypotheses to guide research activities.
- Engagement with community to identify protective factors to balance risk factors and minimize stigmatization potential and refine measures a strength.
- Additional information added about potential role of negative affect to address prior review.
- Use of EMA rather than retrospective methods lacking in context is methodologically advantageous with a combination of signal and event-based prompts.
- Multidimensional assessments of alcohol use and attitudes as well as types of minority stress.
- Variable included positive and negative affect as well as social support, ethnic identity, outness all based on previous literature and/or theory.

Weaknesses

- Sample size of 50 seems small given the number of variables although a power analysis is presented.
- Limitations not clearly presented.

4. Mentor(s), Co-Mentor(s), Consultant(s), Collaborator(s)

Strengths

- Mentor is an experienced alcohol treatment researcher focused on Latinx immigrants
- Co-mentors include experts in adolescent alcohol use, EMA methods, and SMs who are ethnoracial minorities and have existing relationships with mentor with strong publication and funding records.
- Co-mentors, Drs. Colby, Jackson, and Miranda, have significant mentoring experience.

Weaknesses

- Mentoring background of the primary mentor is not explicated in biosketch.
- Addition of very strong biostatistical mentor warranted given the complexity of proposed analyses.

5. Environment and Institutional Commitment to the Candidate

Strengths

- Brown University has a center for alcohol and addiction studies with over 100 affiliated faculty.

- Other supports offered include statistical and methodological consultation.

Weaknesses

- Little to no description of resources available through Brown at large versus the alcohol related center.

Protections for Human Subjects

Acceptable Risks and Adequate Protections

- Protections include minimizing risk of sexual orientation disclosure.

Inclusion Plans

- Sex/Gender: Distribution justified scientifically
- Race/Ethnicity: Distribution justified scientifically
- For NIH-Defined Phase III trials, Plans for valid design and analysis:
- Inclusion/Exclusion Based on Age: Distribution justified scientifically
- Will include both genders, focus on minority youth justified scientifically.

Vertebrate Animals

Not Applicable (No Vertebrate Animals)

Biohazards

Not Applicable (No Biohazards)

Resubmission

- Highly responsive to prior reviews.

Training in the Responsible Conduct of Research

Acceptable

Comments on Format (Required):

- Will attend combination of online courses as well as 7-week RCR series in addition to discussing ethics as part of mentoring meetings along with guided reading.

Comments on Subject Matter (Required):

- RCR series will cover data acquisition, storage, ethical concerns, as well as ethical issues in research and dissemination specific to alcohol use in Latinx, SMY.

Comments on Faculty Participation (Required; not applicable for mid- and senior-career awards):

- 7-week RCR led by Dean, mentors are all faculty members.

Comments on Duration (Required):

- 7 plus weeks.

Comments on Frequency (Required):

- In addition to weekly training and mentoring sessions, will submit bi-annual reports to mentorship team.

Select Agents

Not Applicable (No Select Agents)

Resource Sharing Plans

Not Applicable (No Relevant Resources)

Authentication of Key Biological and/or Chemical Resources

Not Applicable (No Relevant Resources)

Budget and Period of Support

Recommend as Requested

CRITIQUE 3

Candidate: 3
Career Development Plan/Career Goals /Plan to Provide Mentoring: 4
Research Plan: 5
Mentor(s), Co-Mentor(s), Consultant(s), Collaborator(s): 3
Environment Commitment to the Candidate: 2

Overall Impact

The is a resubmission of K08 application requesting funding for career development and research on the stress and alcohol use among Hispanic sexual minority youth in Boston and Providence (RI) areas. The use of EMA is innovative. The candidate has a PhD in Social Work and is currently examining behaviors among ethno-racial and sexual minorities using secondary data. To date, the candidate has one presentation and no publications related to the proposed topic. The career development plan focuses on proposed training and little description of career development beyond the development of expertise in EMA. The research plan includes three sequential phases, each relying on the success of previous phases. EMA procedures are well described; however, there is little detail provided for Phase 1 and for the process in Phase 2. The mentoring team is very strong and relies on the candidate's current mentor to serve as primary mentor during the K08 award period. The Center for Alcohol and Addiction Studies is supportive of the applicant and provides an appropriate environment.

1. Candidate

Strengths

- PhD in Social Work.
- Postdoctoral research fellowship with focus on alcohol and substance abuse intervention research.
- Since previous review, the applicant is currently examining behaviors among ethnoracial and sexual minorities using secondary data.

Weaknesses

- One presentation and no publications on sexual minorities.
- Limited publication record considering completion of PhD and Postdoctoral Research Fellowship.

2. Career Development Plan/Career Goals & Objectives

Strengths

- Build on current expertise and focus career development on alcohol use among youth and sexual minorities, ecological momentary assessment technique, intensive longitudinal analysis, and culturally adapting measures.
- Plans to submit R34.

Weaknesses

- Little information on career development beyond proposed training.
- Development plan suggests a focus on secondary data analysis and manuscript preparation, which does not suggest innovative approach to career development.
- Training plan would benefit from programs in grant preparation.

3. Research Plan

Strengths

- Addresses problem of alcohol use in sexual minority youth (SMY).
- Use of EMA to study stress and alcohol use among Hispanic SMY is innovative.

Weaknesses

- There is a lack of details on Phase 1 (cognitive interviewing).
- Little description of heterogeneity among Hispanic and white SMY.
- There is no mention of pretesting prior to the pilot test.
- The proposed sample include children and adults (age range of 15-19).
- Lack of details for process for completing Phase 2.

4. Mentor(s), Co-Mentor(s), Consultant(s), Collaborator(s)

Strengths

- Primary and co-mentors have very good track record with research and mentoring.
- Mentor roles and frequency of contact are clear.

Weaknesses

- Primary mentor (Dr. Lee) extends current relationship with the applicant.

5. Environment and Institutional Commitment to the Candidate

Strengths

- K award activities will be performed at Brown University’s Center for Alcohol and Addiction Studies and provides reasonable resources.

- Commitment for hire from Institution upon completion of the K or in the event that K award is not funded.
- Overall, very good research environment.

Weaknesses

- None noted.

Protections for Human Subjects

Acceptable Risks and Adequate Protections

- Appropriate as written.

Data and Safety Monitoring Plan (Applicable for Clinical Trials Only):

Not Applicable (No Clinical Trials)

Inclusion Plans Applicable Only for Human Subjects research and not IRB Exemption #4.

- Sex/Gender: Distribution justified scientifically
- Race/Ethnicity: Distribution justified scientifically
- For NIH-Defined Phase III trials, Plans for valid design and analysis: Not applicable
- Inclusion/Exclusion Based on Age: Distribution not justified scientifically
- Not clear why include both children and adults (ages 15-19).

Vertebrate Animals

Not Applicable (No Vertebrate Animals)

Biohazards

Not Applicable (No Biohazards)

Resubmission

- Somewhat responsive to original reviewer comments.

Training in the Responsible Conduct of Research

Acceptable

Comments on Format (Required):

- Appropriate as written.

Comments on Subject Matter (Required):

- Appropriate as written.

Comments on Faculty Participation (Required; not applicable for mid- and senior-career awards):

- Appropriate as written.

Comments on Duration (Required):

- Appropriate as written.

Comments on Frequency (Required):

- Appropriate as written.

Select Agents
Not Applicable (No Select Agents)

Resource Sharing Plans
Acceptable
- Appropriate as written.

Authentication of Key Biological and/or Chemical Resources
Not Applicable (No Relevant Resources)

Budget and Period of Support
Recommend as Requested

THE FOLLOWING SECTIONS WERE PREPARED BY THE SCIENTIFIC REVIEW OFFICER TO SUMMARIZE THE OUTCOME OF DISCUSSIONS OF THE REVIEW COMMITTEE, OR REVIEWERS' WRITTEN CRITIQUES, ON THE FOLLOWING ISSUES:

PROTECTION OF HUMAN SUBJECTS: ACCEPTABLE

INCLUSION OF WOMEN PLAN: ACCEPTABLE

INCLUSION OF MINORITIES PLAN: ACCEPTABLE

INCLUSION ACROSS THE LIFESPAN: ACCEPTABLE

COMMITTEE BUDGET RECOMMENDATIONS: The budget was recommended as requested.

Footnotes for 1 K08 MD015289-01A1; PI Name: Rosales, Robert

NIH has modified its policy regarding the receipt of resubmissions (amended applications).See Guide Notice NOT-OD-18-197 at https://grants.nih.gov/grants/guide/notice-files/NOT-OD-18-197.html. The impact/priority score is calculated after discussion of an application by averaging the overall scores (1-9) given by all voting reviewers on the committee and multiplying by 10. The criterion scores are submitted prior to the meeting by the individual reviewers assigned to an application, and are not discussed specifically at the review meeting or calculated into the overall impact score. Some applications also receive a percentile ranking. For details on the review process, see http://grants.nih.gov/grants/peer_review_process.htm#scoring.

MEETING ROSTER

National Institute on Minority Health and Health Disparities Special Emphasis Panel
NATIONAL INSTITUTE ON MINORITY HEALTH AND HEALTH DISPARITIES
NIMHD Research Career Development Awards (Ks)

ZMD1 XLN (J1)
11/02/2020

Notice of NIH Policy to All Applicants: Meeting rosters are provided for information purposes only. Applicant investigators and institutional officials must not communicate directly with study section members about an application before or after the review. Failure to observe this policy will create a serious breach of integrity in the peer review process, and may lead to actions outlined in NOT-OD-14-073 at https://grants.nih.gov/grants/guide/notice-files/NOT-OD-14-073.html and NOT-OD-15-106 at https://grants.nih.gov/grants/guide/notice-files/NOT-OD-15-106.html, including removal of the application from immediate review.

CHAIRPERSON(S)

FANG, CAROLYN Y, PHD
PROFESSOR AND CO-LEADER
CANCER PREVENTION AND CONTROL PROGRAM
FOX CHASE CANCER CENTER
PHILADELPHIA, PA 19111

SIMON, MELISSA A., MD, MPH
PROFESSOR AND VICE CHAIR OF CLINICAL RESEARCH
DEPARTMENT OF OBSTETRICS AND GYNECOLOGY
AND PREVENTATIVE MEDICINE
FEINBERG SCHOOL OF MEDICINE
NORTHWESTERN UNIVERSITY
CHICAGO, IL 60611

MEMBERS

BOYNTON-JARRETT, RENEE DANIELLE, MD, SCD
ASSOCIATE PROFESSOR
DEPARTMENT OF PEDIATRICS
SCHOOL OF MEDICINE
BOSTON UNIVERSITY
BOSTON, MA 02118

DILLARD, DENISE A, PHD
DIRECTOR OF RESEARCH
DATA SERVICES AND RESEARCH DEPARTMENT
SOUTHCENTRAL FOUNDATION
ANCHORAGE, AK 99508

FRYE, VICTORIA, DRPH
MEDICAL PROFESSOR
DEPARTMENT OF COMMUNITY HEALTH
AND SOCIAL MEDICINE
SCHOOL OF MEDICINE
THE CITY UNIVERSITY OF NEW YORK
NEW YORK, NY 10035

HAN, HAE-RA, PHD, RN
ISABEL HAMPTON ROBB PROFESSOR
SCHOOL OF NURSING
JOHNS HOPKINS UNIVERSITY
BALTIMORE, MD 21205

HARRINGTON, CHERISE BALDWIN, PHD, MPH
ASSOCIATE PROFESSOR
DEPARTMENT OF PUBLIC HEALTH EDUCATION
NORTH CAROLINA CENTRAL UNIVERSITY
DURHAM, NC 27707

JARRÍN MONTANER, OLGA F., PHD, RN
ASSISTANT PROFESSOR
DIVISION OF NURSING SCIENCE
RUTGERS SCHOOL OF NURSING
RUTGERS, THE STATE UNIVERSITY OF NEW JERSEY
NEW BRUNSWICK, NJ 08901

LINK, BRUCE G, PHD
DISTINGUISHED PROFESSOR OF PUBLIC POLICY AND SOCIOLOGY
SCHOOL OF PUBLIC POLICY
UNIVERSITY OF CALIFORNIA, RIVERSIDE
RIVERSIDE, CA 92521

MRUG, SYLVIE, PHD
PROFESSOR AND CHAIR
DEPARTMENT OF PSYCHOLOGY
COLLEGE OF ARTS AND SCIENCES
THE UNIVERSITY OF ALABAMA AT BIRMINGHAM
BIRMINGHAM, AL 35294

SANTOS, HUDSON, PHD, RN
ASSISTANT PROFESSOR
PHD DIVISION
SCHOOL OF NURSING
THE UNIVERSITY OF NORTH CAROLINA AT CHAPEL HILL
CHAPEL HILL, NC 27599

SENTELL, TETINE L, PHD
DIRECTOR AND PROFESSOR
OFFICE OF PUBLIC HEALTH STUDIES
UNIVERSITY OF HAWAII AT MANOA
HONOLULU, HI 96822

SHARKEY, JOSEPH R, PHD, MPH
PROFESSOR
DEPARTMENT OF HEALTH PROMOTION AND COMMUNITY
HEALTH SCIENCES
SCHOOL OF PUBLIC HEALTH
TEXAS A&M UNIVERSITY
COLLEGE STATION, TX 77843

SORKIN, DARA H, PHD
ASSOCIATE PROFESSOR
DEPARTMENT OF MEDICINE
DIVISION OF GENERAL INTERNAL MEDICINE
UNIVERSITY OF CALIFORNIA, IRVINE
IRVINE, CA 92697

SCIENTIFIC REVIEW OFFICER

NAN, XINLI, MD, PHD
SCIENTIFIC REVIEW OFFICER
SCIENTIFIC REVIEW BRANCH
OFFICE OF EXTRAMURAL RESEARCH ADMINISTRATION
NATIONAL INSTITUTE ON MINORITY HEALTH AND
HEALTH DISPARITIES, NATIONAL INSTITUTES OF HEALTH
BETHESDA, MD 20892

EXTRAMURAL SUPPORT ASSISTANT

DAVIS, CRYSTAL
SUPPORT ASSISTANT
SCIENTIFIC REVIEW BRANCH
OFFICE OF EXTRAMURAL RESEARCH ADMINISTRATION
NATIONAL INSTITUTE ON MINORITY HEALTH AND
HEALTH DISPARITIES, NATIONAL INSTITUTES OF HEALTH
BETHESDA, MD 20892

Consultants are required to absent themselves from the room during the review of any application if their presence would constitute or appear to constitute a conflict of interest.
